# Autism Spectrum Disorders and Identified Toxic Land Fills: Co-Occurrence Across States

**DOI:** 10.4137/EHI.S830

**Published:** 2008-08-20

**Authors:** Xue Ming, Michael Brimacombe, Joanne H. Malek, Nisha Jani, George C. Wagner

**Affiliations:** 1 Departments of Neurosciences and Pediatrics, UMDNJ-New Jersey Medical School, Newark, NJ; 2 Department of Preventive Medicine, UMDNJ-New Jersey Medical School, Newark, NJ; 3 Department of Psychology, Rutgers University, New Brunswick, NJ

**Keywords:** autism spectrum disorders, environmental toxins, superfund sites, toxic landfills

## Abstract

It is believed that gene by environmental interactions contribute to the pathogenesis of autism spectrum disorders (ASD). We hypothesize that ASD are associated with early and repeated exposures to any of a number of toxicants or mixtures of toxicants. It is the cumulative effects of these repeated exposures acting upon genetically susceptible individuals that lead to the phenotypes of ASD. We report our initial observations of a considerable overlap of identified toxic landfills in the State of New Jersey and the residence of an ASD cohort, and a correlation between the identified toxic Superfund sites on each U.S. state and the total number of diagnosed cases of ASD in those states. The residence of 495 ASD patients in New Jersey by zip code and the toxic landfill sites were plotted on a map of Northern New Jersey. The area of highest ASD cases coincides with the highest density of toxic landfill sites while the area with lowest ASD cases has the lowest density of toxic landfill sites. Furthermore, the number of toxic Superfund sites and autism rate across 49 of the 50 states shows a statistically significant correlation (i.e. the number of identified superfund sites correlates with the rate of autism per 1000 residents in 49 of the states (p = 0.015; excluding the state of Oregon). These significant observations call for further organized studies to elucidate possible role(s) of environmental toxicants contributing to the pathogenesis of ASD.

## Commentaries

The etiology of autism spectrum disorders (ASD) remains unknown but is thought to involve early exposure to neurotoxicants acting upon genetically susceptible individuals. This gene by environment interaction is believed to affect neural development and lead to the behavioral phenotype of ASD characterized by impaired social reciprocity and communication as well as restricted interests and behaviors. The list of potential neurotoxicants as candidates is long and includes metals, solvents, herbicides, pesticides, and drugs. Likewise, the list of gene candidates is long and includes those involved in neuronal development, neurotransmitter synthesis and degradation, toxicant metabolism, and the management of reactive oxygen species. No single toxicant and no single gene alteration have been identified as causal and, indeed, it may be naive to conclude that such would be the case. We now hypothesize that ASD are associated with early and repeated exposures to any of a number of toxicants or mixtures of toxicants, and that it is the cumulative effects of these repeated exposures acting upon genetically susceptible individuals that leads to the phenotypes of ASD.

To date, most studies assessing the etiology of ASD have examined the body burden of one or more toxicants in the affected individual and/or the mother of the affected individual. With very few exceptions, the limitations of such studies include failure to identify dose, frequency of exposure(s), and timing of exposure(s) to the various toxicants. In addition, the general mobility of families makes it difficult to identify the local environment during which the critical exposure(s) might have occurred.

Nevertheless, Palmer et al. (2006) found a significant increase in the rates of special education students and autism rates associated with increases in environmentally released mercury in the State of Texas (U.S.A). Furthermore, Palmer et al. (2008) found, in the same state, that there was correlation between the increase in autism rate and power plant emission. An independent inverse association of distance of residence to the industrial or power plant emission sources and the rate of autism was reported. Another study suggested a potential association between autism and estimated metal concentrations, and possibly solvents in ambient air in children who were born in the San Francisco Bay area in 1994 (Windham et al. 2006). We now report our initial observation of a considerable overlap of identified toxic landfills in the State of New Jersey and the residence of an ASD cohort currently under the care of the Pediatric Neurologist (XM). On further investigation, we were able to correlate a list of identified toxic Superfund sites on a state-by-state basis together with the total number of diagnosed cases of ASD, again on a state-by-state basis. Here again, we see a considerable overlap and, of importance, this overlap is stronger for the relationship of ASD than for the total population. Since living in the near proximity of either toxic landfills or superfund sites will lead to repeated exposures to mixtures of toxicants, we believe these observations provide general support for our hypothesis noted above.

**Figure 1.**
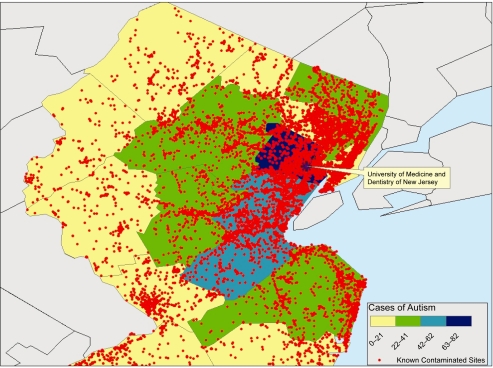
The Distribution of Toxic Landfill Sites and the ASD Cases by Zip Code. From: http://www.state.nj.us/dep/srp/kcs-nj/The number of the autism spectrum disorders cases in a specific county is color coded according to the density scale in the insert. These data represent the cases seen at the Autism Center from 1998 to 2006, not population based prevalence. Each red dot represents a toxic landfill site.

Participants in the initial observation were children with ASD derived from two groups. The first group was 495 autistic patients evaluated by the pediatric neurologist from 1998 to 2006 through the Autism Center at UMDNJ-New Jersey Medical School. The diagnosis of ASD (autism, pervasive developmental disorder-not otherwise specified or Asperger’s syndrome) was made or confirmed based on DSM-IV criteria. Autism Diagnostic Interview-Revised, Autism Diagnostic Observation Schedule-Generic, and/or Childhood Autism Rating Scale were used for confirmation of diagnosis in approximately 6% of the children who were/are participating in other funded biological or genetic research projects. There were 372 males and 123 females in this group. The age range of the subjects at time of analysis was 4 to 25 year old. Among the 495 patients, there were 198 patients with autistic disorder, 252 patients with pervasive developmental disorder-not otherwise specified, and 45 patients with Asperger’s syndrome. The residence of these 495 patients in New Jersey by zip code was plotted on a map of Northern New Jersey by color code of ASD case density. The toxic landfill sites in Northern New Jersey were then plotted on the same map (red dots; Fig. 1, data from http://www.state.nj.us/dep/srp/kcs-nj/). Significant correlation was found between the number of cases and the number of toxic landfill sites (p = 0.019). The area of highest ASD cases coincides with the highest density of toxic landfill sites while the area with lowest ASD cases has the lowest density of toxic landfill sites. One apparent exception for this relationship is the New Jersey area bordering New York City. The possible explanation for the low number of ASD cases seen in our center from this particular area with a high density of toxic landfill sites may be that the ASD cases from this area are cared for in the medical centers of New York City.

**Figure 2.**
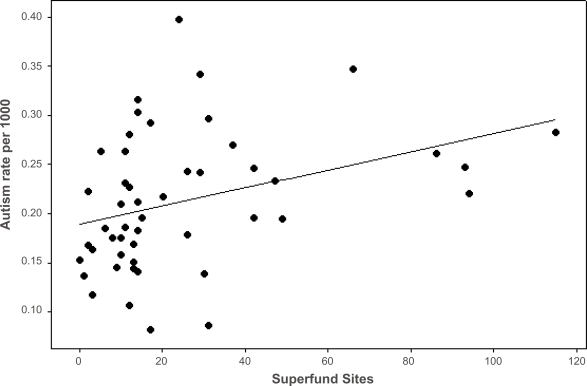
The Correlation between EPA Superfund Sites and Autism Rate in the U.S. States. Pearson correlation of EPA Superfund Sites and Autism rate per 1000 = 0.345. P-Value = 0.015. Each dot represents a different state except Oregon. S = 0.0658, R-Sq = 11.9%, R-Sq (adj) = 10.0%.

Our second strategy was to examine the list of Superfund sites on a state-by-state basis as identified by the Environmental Protection Agency (EPA) in 2006 {www.epa.gov/Superfund/sites/npl/npl.htm}. This list was then compared to the total population of that state from the latest 2000 census {www.census.gov} and finally with the number of identified cases of autism as compiled by the U.S. Individuals With Disabilities Education Act data in 2006 {www.IDEAdata.org}. The EPA Superfund sites and autism rate per 1000 in the 50 states are shown in Table 1. The data were subjected to analyses by means of simple regression and correlation. Figure 2 shows that there is a statistically significant correlation between the number of identified superfund sites and the rate of autism per 1000 population in 49 U.S. states (p = 0.015; Oregon was excluded). Oregon is a statistical outlier in this state-by-state correlation as it appears to have the highest autism rate (0.648 autism case per 1000 population) and a relatively low number of superfund sites (11 sites). As noted, the correlation between autism rate and the number of superfund sites exceeds that between the correlation between state population and autism rate.

These observations reveal considerable overlap between identified toxic landfills and diagnosed cases of autism/ASD. As with any correlation analysis, it is not possible to conclude the relationship is causal. However, the strength of the correlation and the fact that autism cases related to the number of Superfund sites is consistent with the hypothesis that the etiology of autism/ASD, in part, is dependent upon early and repeated exposure to mixtures of environmental toxicants. The NJ data was not population based and may have been subject to referral bias due to the location of the autism center. In addition, the general mobility of families in and out of the area may have been a confounding factor. Nonetheless, these significant observations provide the basis for further organized studies to elucidate role(s) of environmental toxicants contributing to the pathogenesis of ASD.

## Figures and Tables

**Table 1.** Autism Rate and EPA Superfund Sites of 50 States.

**States**	**EPA Superfund Sites^1^**	**Autism Incidence^2^**	**Population^3^**	**Autism rate per 1000**
Alabama	13	670	4,447,100	0.150659981
Alaska	5	165	626,932	0.263186438
Arizona	8	897	5,130,632	0.174832262
Arkansas	10	560	2,673,400	0.209471086
California	93	8,376	33,871,648	0.247286462
Colorado	17	350	4,301,261	0.081371486
Connecticut	14	1,032	3,405,565	0.303033417
Delaware	14	248	783,600	0.316488004
Florida	49	3,114	15,982,378	0.194839591
Georgia	15	1,602	8,186,453	0.195689146
Hawaii	3	198	1,211,537	0.163428769
Idaho	6	239	1,293,953	0.184705318
Illinois	42	2,435	12,419,293	0.196065911
Indiana	29	2,080	6,080,485	0.342077976
Iowa	11	543	2,926,324	0.185557033
Kansas	10	471	2,688,418	0.17519597
Kentucky	14	739	4,041,769	0.182840731
Louisiana	11	1,032	4,468,976	0.230925384
Maine	12	358	1,274,923	0.280801272
Maryland	17	1,551	5,296,486	0.292835665
Massachusetts	31	543	6,349,097	0.085523973
Michigan	66	3,449	9,938,444	0.347036216
Minnesota	24	1,958	4,919,479	0.398009627
Mississippi	3	333	2,844,658	0.117061524
Missouri	26	1,361	5,595,211	0.243243731
Montana	14	127	902,195	0.140767794
Nebraska	13	289	1,711,263	0.168881113
Nevada	1	273	1,998,257	0.136619064
New Hampshire	20	268	1,235,786	0.216866027
New Jersey	115	2,378	8,414,350	0.282612442
New Mexico	12	193	1,819,046	0.106099571
New York	86	4,951	18,976,457	0.260902233
North Carolina	31	2,391	8,049,313	0.297043984
North Dakota	0	98	642,200	0.152600436
Ohio	30	1,574	11,353,140	0.138640059
Oklahoma	10	547	3,450,654	0.158520675
Oregon	11	2,218	3,421,399	0.648272826
Pennsylvania	94	2,707	12,281,054	0.220420821
Rhode Island	12	238	1,048,319	0.227030131
South Carolina	26	717	4,012,012	0.178713324
South Dakota	2	168	754,844	0.222562543
Tennessee	13	819	5,689,283	0.143954871
Texas	42	5,134	20,851,820	0.24621352
Utah	14	472	2,233,169	0.211358836
Vermont	11	160	608,827	0.262800434
Virginia	29	1,714	7,078,515	0.242141184
Washington	47	1,376	5,894,121	0.233452961
West Virginia	9	262	1,808,344	0.144883938
Wisconsin	37	1,445	5,363,675	0.269404839
Wyoming	2	83	493,782	0.168090372

**Sources:** 1. www.epa.gov/superfund/sites 2. U.S. Individuals With Disabilities Education Act data: http://www.IDEAdata.org, Specifics on whether the diagnosis of “Autism” indicates “Autistic Disorder” or “Autism Spectrum Disorders” is not available. 3. www.census.gov
